# Intelligent Selection Algorithm of Optimal Logistics Distribution Path Based on Supply Chain Technology

**DOI:** 10.1155/2022/9955726

**Published:** 2022-04-14

**Authors:** Dan He

**Affiliations:** Zhejiang Industry & Trade Vocational College, Wenzhou 325003, Zhengjiang, China

## Abstract

How to realize the intelligence of logistics distribution is a hot research topic at present. How to reasonably allocate vehicles, optimize driving routes and travel time, deliver goods to customers on time at the lowest cost, and realize efficient and low-cost operation of the logistics distribution system has always been a problem in academia and industry for many years. Logistics enterprises face problems such as low efficiency of logistics operation, lack of scientific rationality of logistics resource planning, and lack of overall optimization of logistics management operation mode. These are severe tests that steel companies must accept. Under the background of logistics supply chain, the integrated service platform of logistics supply chain has become an urgent research topic. This study takes a steel enterprise as the main research background. On this basis, the two core modules of warehousing and distribution in the logistics business of iron and steel enterprises are qualitatively analyzed, the concept of business process reengineering is proposed, and the logistics supply chain of iron and steel enterprises is established. The concept of comprehensive service platform is realized through RFID technology. In addition, this study conducts a comprehensive analysis and research on the logistics distribution path optimization and vehicle scheduling problem, designs and implements a logistics vehicle scheduling management system, and then adopts the multiobjective method to solve the logistics distribution path planning problem, SMEs. Genetic algorithm and a simulation decision-making subsystem suitable for this problem are designed, which can better solve the problem of route optimization and vehicle scheduling in small-scale distribution.

## 1. Introduction

The relationship between logistics and economic activities is two-way. The existence and development of economic activities stimulate the demand for logistics services, resulting in new logistics facilities and the transformation of existing facilities; without the support of modern logistics systems, economic activities are difficult to smooth operation, and the development of logistics can stimulate the development of economy and the connection and cooperation between regions. Therefore, the two are interdependent and interact [1–3]. With the development of human society to this day, more and more attention has been paid to sustainable development, which makes population, economy, society, environment, and resources coordinate with each other, and the development model that can meet the needs of the contemporary people and the needs of future generations has become an economic growth mode. This also makes the rationalized demand model based on the rational use of the environment and resources as a new development trend. In the future, the demand for urban logistics will develop in a direction that is conducive to environmental protection and rational utilization of resources [4, 5]. This changing trend is manifested.

1. Human beings will become more restrained in their demands for natural resources, especially energy consumption and waste discharge will be kept to a minimum as much as possible; the transportation volume and transportation methods of various materials will be affected under the premise of economy and environmental protection strictly constrained [6–9].
2. The spatial and temporal distribution of logistics demand will be more rational. It is not only beneficial to the main body of logistics demand (minimum time, energy and economic effort, maximizing utility) but also beneficial to the society (environmental protection, logistics social cost saving, and resource consumption saving). When meeting logistics needs, we will choose more clean, low energy consumption logistics organization methods, choose noncongested routes and areas, and avoid peak hours of logistics travel as much as possible [10, 11].

For basic needs, in addition to reasonable constraints on themselves (abandoning unnecessary logistics activities and rationally choosing the method, route, and time of logistics activities), people will also take more scientific means to compress basic needs. These means and measures are all-round: scientifically organize urban space; arrange various urban functional facilities (residential, commercial, public services, and cultural education); improve the production technology, production environment, and production (work) methods of various industries; reduce the degree of spatial aggregation of people and things, so that many production operations and business processing can be scattered in each demand source or nearby relatively concentrated places; and provide the most human-friendly and most resource-saving communication methods and shopping consumption [12–14]. With the further deepening of logistics theoretical research, the continuous optimization of logistics organization models, the continuous emergence of new logistics technologies, and the further promotion of various management concepts, the derived logistics needs will be more rational, and these advanced science and technology will be rationalized. The demand provides guarantee conditions, so that people are satisfied with the satisfaction of demand, the logistics service reaches a higher level, and the logistics supply and logistics demand will reach a higher-quality balance point. To sum up, the development of modern science and technology has created necessary conditions for the rationalization of urban traffic demand and logistics demand [15–17].

Logistics activities affect urban traffic, such as traffic congestion, negative impact on the environment, and energy consumption. Therefore, it is necessary to quantitatively analyze logistics activities, logistics demands, and the social impact of logistics activities by applying demand-supply models. However, most such studies are currently aimed at travelers, and there are few studies on the flow of goods. It is generally believed that in urban planning, traffic congestion is a very prominent problem, and logistics activities have a greater impact on traffic than the activities of travelers. This may be because there are many factors affecting the transportation of goods. Considering the transportation problem of freight flow, modeling is difficult, and the high complexity of the urban logistics system lies in the decision makers, including private car owners, fleets, and many factors. In addition, the predictions of the logistics system are also very different. The core of such models is to predict the activities of logistics companies. The economic subjects of the logistics system include producers, consumers, carriers, potential carriers, and the government. Activity is influenced by the interaction between policymakers and this economic agent [18, 19]. In a general sense, logistics demand forecasting is to use a certain forecasting method or forecasting model for a given area under a certain social and economic environment, according to the current logistics demand and supply situation, and according to the future social and economic development plan or forecast and change trends of production and consumption patterns, analyze and calculate the logistics demand in each characteristic year in the planning area and obtain the social macrologistics flow, distribution of logistics demand, and logistics operation modes (transportation, warehousing, and other logistics operation elements), the flow of goods and the flow of goods undertaken by each node in the logistics network and the facilities in the path.

**Figure 1:**
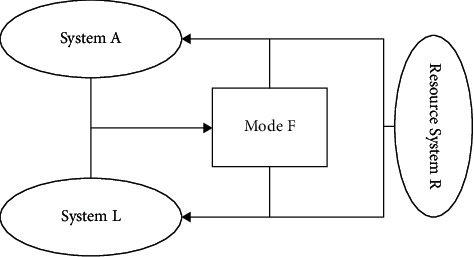
The role of the logistics system.

Judging from the existing logistics forecasting research at home and abroad, the commonly used forecasting methods include regression analysis, exponential smoothing, Markov analysis, time series model, grey model, and input-output analysis, most of which belong to trend extrapolation and causal analysis. The logistics system is a very complex system. When analyzing the logistics system, it is necessary to apply the framework of the system analysis method to analyze the logistics system and the social activity system as a whole. The object system is composed of the logistics system L, the social activity system A, and the logistics organization mode F, and the resource and policy system *R* is an external system closely related to the object system. From the point of view of economics, the logistics system L is the supply side, the social activity system A is the demand side of logistics services, and the logistics mode F, which represents the logistics demand on the logistics network, is the result of the balance of logistics services in the logistics market. The four systems have the basic relationships as shown in Figure 1.

The overall operation of logistics is based on sharing and collaboration, and the existing logistics management model is difficult to adapt to the requirements of overall operation. Therefore, the supply chain management process has a lot of room for improvement. In view of this, building a logistics supply chain comprehensive service platform that can cover the scope of core business management can achieve the development goals of logistics enterprises and play a major supporting role in the process of enhancing core competitiveness. This study conducts a comprehensive analysis and research on the logistics vehicle scheduling and distribution path optimization problem. First, the logistics vehicle scheduling management subsystem is designed and implemented. Then, in the process of solving the problem of path planning faced by small and medium-sized enterprises, the multiobjective method is adopted. The genetic algorithm and designing a simulation decision-making subsystem are suitable for this problem, which can better solve the problem of route optimization and vehicle scheduling in small-scale distribution.

This study is divided into five sections. The first section mainly introduces the research background and demand, supply chain management, business process reengineering, information technology, and other related concepts and the significance of the research. The current situation is described and analyzed, and the research objectives and content of this study are clarified. The second section analyzes the demand of an iron and steel enterprise for logistics supply chain management and comprehensively analyzes the current situation, problems, and bottlenecks faced by an iron and steel enterprise in logistics supply chain management. The third section is the technical research of logistics supply chain management of an iron and steel enterprise, including the design and layout, planning and control of logistics supply chain management, and its operation management. The fourth section is the architecture and application of the iron and steel logistics supply chain service platform, including logical architecture, business architecture, application background introduction, application process and results, and application benefit analysis. The fifth section is summary and prospect, which summarizes the research conclusions of this study, and looks forward to the deficiencies of this study and the follow-up research direction.

The research contributions of this study are as follows:

1. This study takes a steel enterprise as the main research background. On this basis, the two core modules of warehousing and distribution in the logistics business of iron and steel enterprises are qualitatively analyzed, the concept of business process reengineering is proposed, and the logistics supply chain of iron and steel enterprises is established.
2. The study proposes that the concept of using the integrated service platform is realized through RFID technology
3. This study conducts a comprehensive analysis and research on the logistics distribution route optimization and vehicle scheduling problem, designs and implements a logistics vehicle scheduling management system, and then adopts the multiobjective method to solve the logistics distribution route planning problem.

## 2. Demand Analysis of Logistics Supply Chain Management

### 2.1. Current Situation and Bottleneck of Logistics Supply Chain Management

This study conducts a comprehensive analysis and research on the logistics distribution path optimization and vehicle scheduling problem, designs and implements a logistics vehicle scheduling management system, and then adopts the multiobjective method to solve the logistics distribution path planning problem. At present, there are four major problems in logistics:

The degree of logistics standardization is not high, and the business standards are not uniform. On the one hand, there is no unified logistics standard (especially production logistics), and there are inconsistencies in the understanding, operation, and management of logistics, which also affect the overall accounting of logistics costs. On the other hand, external logistics service providers, especially ports, stations, docks, and yards all over the country, have great differences in operational standards, and specific operational levels such as transportation, hoisting, and warehousing, which hinders the standardization and standardized operation of logistics, can be defined as

(1) $$\begin{array}{c} \min\underset{x\in \Omega }{F\left(x\right)}\left(f_{1}\left(x\right),f_{2}\left(x\right),\ldots ,f_{M}\left(x\right)\right), \end{array}$$

where *x* = (*x*_1_, *x*_2_, ..., *x*_N_) is an *N*-dimensional space vector, *n* is the feasible solution space, and *f*_1_(*x*), *f*_2_(*x*),…, *f*_M_(*x*) are the *M* objective functions.

The integration level of logistics information management is not high, and it is difficult to collect and process information. The logistics business of an iron and steel enterprise includes a series of logistics businesses such as raw material logistics, production logistics, and sales logistics and uses the corresponding Bao steel Co., Ltd. raw material procurement logistics control system, manufacturing MES system, and sales logistics control system; however, due to the decentralized configuration, it is difficult for the company to fully grasp the logistics information. The logistics infrastructure lacks unified master plan management and planning. The specific manifestations are as follows: on the one hand, the logistics infrastructure of the existing base has not been fully shared, and on the other hand, according to the planning requirements, there are gaps in the logistics resources such as warehouses of each unit to varying degrees.

(2) $$\begin{array}{c} \left\{\begin{array}{c} \min Y_{1}=\sum_{k\in V}\sum_{i\in C}\sum_{j\in C}c_{ij}X_{ijk}\\ \min Y_{2}=\sum_{k\in V}\sum_{j\in C}X_{0jk} \end{array}\right. \end{array}$$

When the transportation capacity is tight, the transportation guarantee is insufficient, and the logistics cost cannot be effectively controlled. The company does not have enough control over ports and shipping capacity. Once the market is tense, there will be insufficient loading and unloading capacity or shipping capacity, and the timely loading, unloading, and shipping of raw materials and finished products cannot be guaranteed; this can be defined as

(3) $$\begin{array}{c} hk=\frac{\sum_{r=1}^{p}u_{r}y_{rk}}{\sum_{i=1}^{m}v_{i}x_{ik}}\left(v\geq 0,u\geq 0\right). \end{array}$$

Therefore, the existing logistics management mode is difficult to meet the needs of the overall logistics operation of the leap-forward development. On the whole, the efficiency of logistics operation is low, the logistics resources lack scientific and reasonable overall planning, and there is still a large space for integration and optimization of logistics management and operation mode.

### 2.2. Comprehensive Analysis of Supply Chain Management

The logistics business must provide full-process integrated value-added services in terms of specialized, all-round, full-process, and differentiated services. Starting from the core concept of creating value through services, create an information-oriented electronic technology as a means to integrate various supply chains. The logistics information system of the link realizes the controllability of the entire logistics link and improves the lean operation level of logistics, and the system can be shown as

(4) $$\begin{array}{c} hk=\frac{\sum_{i=1}^{p}u_{r}y_{rk}}{\sum_{i=1}^{m}v_{i}x_{ik}}\leq 1. \end{array}$$

The effective integration of business and logistics resources is the necessary prerequisite and foundation for the expansion and refinement of the iron and steel logistics business, and it is also one of the important means of cost control. The continued growth of third-party logistics companies' profits depends largely on the formation of economies of scale. Iron and steel logistics enterprises are currently in the period of integration of business and resources, and the integration of business and resources is an important means and measure for the formation of scale effects and the expansion and refinement of business. We can know that the traditional functional organizational structure has been unable to adapt to the new market external environment dominated by “customers, competition, and changes” because it artificially separates the integrity of the business process, which leads to the information transmission in the supply chain process. The following equation shows a process of supply chain transfer and the transfer method of different parameters.

(5) $$\begin{array}{c} \text{max}\frac{\sum_{i=1}^{p}u_{r}y_{rk}}{\sum_{i=1}^{m}v_{i}x_{ik}},v\geq 0,u\geq 0,i=1,2,\ldots ,p;j=1,2,\ldots ,n. \end{array}$$

Distortion and delay and failure of information communication destroy the objective basis of performance evaluation. The organizational structure of iron and steel logistics enterprises is evolved from the traditional functional organizational structure, and in essence, there is no reasonable arrangement according to the business process. The main content of the logistics supply chain business process engineering is to reform and transform the original organizational structure on the basis of the process. For enterprises, it is of great significance to use the opportunity of enterprise logistics supply chain process engineering to readjust the functional organizational structure of the original supply chain-related departments.

(6) $$\begin{array}{c} \text{max}\sum_{\text{r}=1}^{p}u_{r}y_{rk}. \end{array}$$

Subject to

(7) $$\begin{array}{c} \sum_{r=1}^{p}u_{r}y_{rj}-\sum_{i=1}^{m}V_{i}y_{ij}\leq 0,\\ \sum_{\text{r}=1}^{m}V_{i}y_{ik}=1,\;V\geq 0,\;u\geq 0,\;i=1,\;2,\ldots ,\;p;\;j=1,\;2,\;\ldots ,\;n. \end{array}$$

## 3. Methodology

Logistics allocation is an important part of logistics demand forecasting, which is to allocate the L-OD volume between logistics subregions to the logistics network. The accuracy of the allocation will determine whether it can truly reflect the changing trend of the logistics network nodes and the logistics flow on the path during the planning period and provide a basis for the logistics network evaluation planning and quantitative analysis. The order of construction projects, construction scale, and investment in the logistics system planning are also determined by the logistics demand on the logistics network and reflect the supply service level of the logistics network and the quality of the use of the logistics network. At the same time, logistics distribution is also the core content of the feasibility study of logistics infrastructure construction projects. It is the premise and basis for evaluating the logistics operation status and comprehensively analyzing the necessity and feasibility of construction projects. The main basis for project construction planning and economic evaluation is.

(8) $$\begin{array}{c} q_{ij}^{k}=a_{i}^{k}o_{i}^{j}\beta_{j}^{k}D_{j}^{k}\exp\left(-\gamma^{k}C_{ij}^{k}\right). \end{array}$$

When the logistics system provides services, there are various forms. According to the form of facilities, there are lines, stations, and warehouses, channel capability, etc. and services (speed, convenience, reliability, and known ability). The choice of the logistics chain mainly involves the value orientation of the owner and the customer, as well as the technical and economic characteristics of the logistics chain. The logistics chain selection forecast is mainly based on the measurable principle to predict the selection and combination of the logistics chain and its organization methods in the future characteristic years of the planning area. The amount of logistics needs to be undertaken. The probit model, multinomial logit model, and nested logit model were used in prediction. The following mainly studies the factors that affect the choice of cargo transportation mode, summarizes the relevant laws of logistics transportation, and lays the groundwork for logistics distribution prediction as

(9) $$\begin{array}{c} \text{Support}\left(m\right)=\left\{\begin{array}{cc} 0, & \text{load}<\text{threshold}\\ 1, & \text{load}\geq \text{threshold} \end{array}\right). \end{array}$$

When there are multiple alternative modes of transportation between any two points, it is necessary to share the mode of transportation. The essence of choosing a mode of transportation is to choose a service, and the main body of logistics needs to choose the best mode among many modes to transport. The transportation of goods under different conditions has different demand characteristics in terms of transportation time, transportation cost, and transportation service reliability, and different transportation methods have different transportation service attributes due to different technical performance, transportation conditions, and service forms. Therefore, when cargo owners or trips choose different modes of transportation, the degree of satisfaction of their needs is different. By weighing the service attributes of each mode of transportation, the cargo owner (in a broad sense, that is, the decision maker of the logistics mode of transportation or logistics organization mode) finally chooses the mode of transport that can best meet or match their needs.

(10) $$\begin{array}{c} \text{Load\_production}=\frac{{\text{Absolve}}_{\text{unit}}*{\text{Target}}_{\text{pro}}-\text{content}*0.15*\text{revise\_parameters}}{\text{Content}*\text{vilidation}}. \end{array}$$

The characteristics or attributes of the goods to be transported and various characteristics of the goods need to be considered when choosing a transport mode, such as density, packaging size, time constraints, cargo type, value, volume, and weight. Some literature point out that different types of trucks should be considered in different models when analyzing and predicting cargo demand. Cargo operations must consider the characteristics of the cargo, the condition of the driver, and a series of legal and custom constraints. The choice of the model depends more on the characteristics of the goods being transported. Most studies show that there is a big difference between the prediction results of the choice of freight transportation mode and the actual situation, and the use of a model parameter for different types of goods will cause a large deviation. Among the discrete choice models, the logit separation model is widely used in mode choice analysis. The Box–Cox logit model is much superior to the linear logit model, but the application of the model in the transportation domain is not as the following formula given.

(11) $$\begin{array}{c} \varpi_{XY}=\frac{E\left(XY\right)-E\left(X\right)E\left(Y\right)}{\sqrt{E\left(X^{2}\right)-{\left(E\left(X\right)\right)}^{2}\sqrt{E\left(Y^{2}\right)-{\left(E\left(Y\right)\right)}^{2}}}}. \end{array}$$

What route and node a logistics OD chooses to achieve its purpose is determined by the characteristics of the logistics subject (such as starting point, destination, physical characteristics, chemical characteristics, and value orientation), and the technical and economic characteristics of facilities (such as capabilities and services, and economic characteristics) factors interact with each other. The logistics distribution forecast is to predict the various logistics demands on each logistics facility (route and node) in the future characteristic year and obtain the demand assumed by each logistics facility.

## 4. Logistics Distribution Route Selection Technology

In a general logistics network, there are many paths between two logistics nodes. How to correctly and reasonably distribute the L-OD amount to each road between *O* and *D* is the problem to be solved by the logistics distribution model:

(12) $$\begin{array}{c} QS\left(Vi\right)=\left(1-m\right)+m*\sum_{Vj\in \text{In}\left(Vi\right)}\frac{wji}{\sum_{vk\in \text{Out}\left(Vj\right)}wjk}QS\left(Vj\right). \end{array}$$

Logistics allocation is to allocate L-OD of a certain logistics transportation mode in each characteristic year in the planning area to each node and path in the logistics network according to certain rules, so as to find out the logistics on each node and path.

In logistics distribution, the following rules are generally followed: if there are many paths between two points and the logistics exchange between these two points is very small, these logistics flows will obviously flow along the shortest path. With the increase of logistics flow, the logistics distribution volume on the shortest path also increases. When the increase reaches a certain level, the logistics operation time and cost on the shortest path will increase due to congestion, and this part of the logistics demand will choose the second short circuit. As the volume of logistics between the two points continues to increase, all logistics networks between the two points have the potential to be utilized. If users of all routes know exactly the time and cost of each road and choose the shortest path, the time and cost of each route used between the two points will be equal, and the road that is not used will be longer. Or more, this state is the equilibrium state of the road. The research of logistics distribution technology and model in equilibrium state is the focus of this research.

Route selection means that the logistics demand selects an appropriate route from the selectable routes, and logistics distribution is to determine the logistics flow on specific road sections in the network under the given conditions of the total logistics flow between each node of the logistics transportation network. In the distribution of the urban logistics network, there are many factors that affect the choice of logistics route, which is the result of many factors. Not only the driver's experience, physiological factors, and psychological factors will affect the choice of the route but also the road conditions, the nature of the goods in the logistics transportation, and the value orientation of the cargo owner to the logistics transportation all affect the distribution of the logistics flow.

Since there are many factors affecting distribution and the mechanism of action is very complex, it is impossible and unnecessary to establish a unified model including various factors. According to a previous research, time and cost are the two main factors affecting logistics distribution. How to integrate these two factors into the model in logistics distribution is one of the focuses of the study.

The capacity-limited allocation method also allocates the logistics volume between logistics zones to the route with the smallest right of way between logistics zones, but it considers the relationship between time or service cost and logistics volume. When the flow of goods reaches a certain amount, the speed of the vehicle will decrease with the increase of the flow of goods, and the right of way will become larger. Therefore, the route with the smallest right of way will be allocated first, and when the flow of goods reaches a certain amount, the route right is no longer the smallest, and the logistics flow will be allocated to other smallest routes. According to the processing method of the relationship between the right of way and the load, there are two forms of capacity-limited allocation, namely, capacity-limited incremental load allocation and capacity-limited iterative balanced allocation.

(13) $$\begin{array}{c} \text{Bhs}=P_{r}+Q_{h}*R*\frac{T}{\left(16*365\right)}. \end{array}$$

When the logistics distribution model is established on the basis of user balance, that is, users try to choose the shortest path to achieve network balance, so that the time or cost of each route used are equal and minimum, then it is called user balance or user balance (optimal (user equilibrium UE) model). The distribution result of the model established according to this principle should be the result of the user's actual route selection on the logistics network. It can be described by the following model:

(14) $$\begin{array}{c} \min Z\left(X\right)=\sum_{a}\int_{0}^{Xn}t_{a}\left(\omega \right)\text{d}\omega . \end{array}$$

Subject to

(15) $$\begin{array}{c} \sum_{k}f_{k}^{rs}=q_{rs},\forall r,s,\\ f_{k}^{rs}\geq 0\;\forall r,s,\\ Xa=\sum_{r}\sum_{s}\sum_{k}f_{k}^{rs}\delta_{a,k}^{rs},\forall a. \end{array}$$

The second principle reflects a goal, that is, in what way is the best distribution. In the actual network, the state described by the second principle cannot occur unless all logistics service providers cooperate with each other to be the most efficient system for the system. Efforts to optimize and such efforts are impossible. This principle provides a decision-making method for program managers. The model established according to the second principle is called the system optimization model (SO) and can be described by the following model:

(16) $$\begin{array}{c} \min Z\left(X\right)=\sum_{a}x_{a}t_{a}\left(x_{a}\right). \end{array}$$

Subject to

(17) $$\begin{array}{c} \sum_{\text{total}}f_{m}{}^{qs}=F_{rs},\forall m,s,\\ f_{m}{}^{rs}\leq 0\;\forall m,s. \end{array}$$

According to the analysis of the logistics distribution model, it can be seen that it affects the logistics volume in the path and nodes in the logistics network, that is, the time or cost on the road section, and the comprehensive consideration is the logistics space impedance. Logistics space impedance or service quality includes many contents: time, safety, and cost. When studying travel behavior in urban traffic, time is often used as the main measurement standard. When studying cargo transportation, both time and expense are more important. The purpose of studying urban logistics is to develop and implement effective management strategies to reduce social, economic, and environmental costs caused by the use of vehicles to distribute materials in urban areas. Traffic congestion and environmental impact are closely related to impedance (or speed), while fees are also affected by road conditions (e.g., fees are related to road class and load).

(18) $$\begin{array}{c} C_{ij}^{k}=f_{ij}^{k}+\sum_{m}a_{m}^{k}V_{m}^{k}. \end{array}$$

The analysis of shipping rates can be done using a spatial price balance model that applies supply and demand functions to give shipping prices and quantities for transactions between spatially separated markets, while pointing out that certain factors of demand and supply may affect shipping prices between regions.

**Figure 2:**
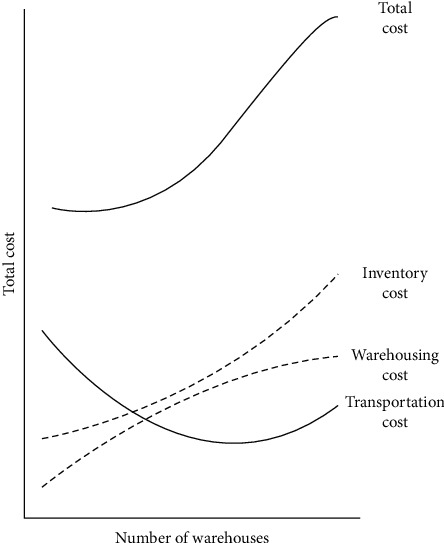
Different cost on supply chain.

As shown in Figure 2, it indicates the cost of different factors of supply chain. The models of logistics distribution can usually be divided into the nonequilibrium model and equilibrium model, as well as the extension of the equilibrium model, including the elastic demand allocation model (variable demand), the random allocation model, the balance model considering the mutual influence of the road segment logistics, and the dynamic allocation model. The random allocation model includes nonequilibrium random allocation (simulation-based, proportion-based) and stochastic user equilibrium. Due to the complexity of the equilibrium state of the logistics network and the difficulty of establishing and solving the equilibrium model, the practical application of the equilibrium model is limited. Therefore, the nonequilibrium model and the equilibrium model are introduced.

Table 1 provides the information of test data. The shortest path logistics distribution method is a static logistics distribution method. This method takes the right of way (traveling time or cost between two nodes) as a constant and also assumes that the speed of the vehicle is taken as the design speed of the road section under the condition of free traffic flow. The L-OD volume of each OD point pair is all distributed on the shortest path connecting an OD point pair, and the other paths do not distribute the material flow. After all, the L-OD quantities of all OD point pairs are allocated to the road network; according to the above principles, the logistics flow of each road section and each logistics node can be accumulated. This method does not consider the limitation of the supply capacity of the road section when distributing the logistics volume or does not consider the logistics distribution phenomenon that the excessive logistics volume will affect the supply level of the logistics service and may choose other routes. It is also called the unrestricted distribution method or all-or-nothing distribution. The shortest path allocation method is simple to calculate, but the distribution results are unreasonable, the distribution is uneven, and the error is larger than the actual situation. But the shortest path allocation method is the basis for other allocation methods.

**Figure 3:**
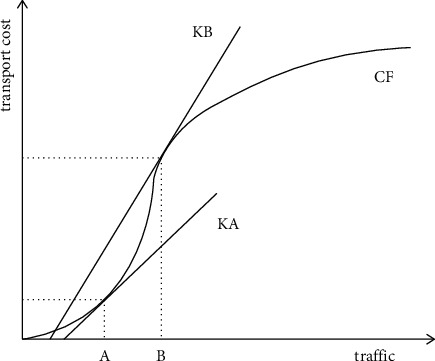
The relation curve between the traffic and transportation cost.

As shown in Figure 3, the curve CF reflects the relationship between the transportation cost and the flow between network nodes, and the slope represents the unit transportation cost. Obviously, the slope KA when the flow is A is greater than the slope KB when the flow is B. Therefore, although the flow is B, the transportation cost of the logistics network is larger than the transportation cost when the flow is A, but the unit transportation cost when the flow is B is smaller than the unit transportation cost when the flow is A. This phenomenon is caused by the scale effect of the hub-and-spoke logistics network. Although, in the hub-and-spoke logistics network, goods are transported through the hub, detours will be caused, and the transportation time and hub construction and operation costs will increase, but the scale effect of the hub-and-spoke logistics network can balance these unfavorable aspects, greater benefit. The larger the scale effect is, the smaller the total cost of hub-and-spoke logistics network operation will be and the greater the benefit will be to the network operator. Therefore, it is necessary to consider the impact of the scale effect when modeling.

## 5. Conclusion

With the process of industrialization and urbanization and the rapid development of information technology, logistics plays an increasingly important role in social production and meeting the needs of life. However, at the current stage of our country, the logistics theory lags behind and hinders the development of the logistics industry, especially the lack of unified standards and theoretical support for logistics system planning. Therefore, it is necessary to study and predict logistics demand, an important part of the logistics system planning complete. As the last stage of logistics demand forecasting, logistics distribution plays an important role in the logistics system planning and provides decision-making basis for the construction of logistics infrastructure; at the same time, in logistics, the research of distribution can optimize the logistics plan and improve the scientificity and rationality of transportation and logistics operations. Therefore, the study of the distribution theory of logistics has positive theoretical and practical meanings.

Modern logistics is developing vigorously in the world, while my country's logistics industry is in a rapid start-up stage. The logistics demand forecasting theory, which is the main content of modern logistics system planning theory, is also in the process of continuous deepening. The logistics distribution problem is one of the research topics with rich connotation and great exploration. Because logistics distribution involves many fields and many disciplines, it is quite complex, especially when it involves a large workload in the process of model establishment and solution, and requires strong mathematical and computer theories. In this dissertation, we only make a certain analysis of the general law, and the quantitative analysis of this problem has not been covered, such as the logistics demand and the nature of land use, the overall layout of the city, and so on. Logistics demand includes the needs of social production and life for the entire logistics operation process, such as packaging, loading and unloading, storage, and circulation processing. Correspondingly, logistics demand forecasting should also include many aspects. In this study, we only use the relevant theories of traffic demand analysis and forecasting to explore and conduct preliminary research on logistics distribution in logistics demand forecasting. Therefore, the research on demand forecasting in other fields of logistics operations needs to be further deepened. Logistics distribution is an extremely complex subject, involving various factors. This study only analyzes the time and cost to establish a distribution model; at the same time, there are certain assumptions in the establishment of the model, so it is necessary to make assumptions about the assumptions. Carry out in-depth research, such as the relationship between price and demand, supply and service cost, and logistics workload and internal mechanism. At the same time, the parameter calibration method of the model and the data collection method need to be further studied.

## Figures and Tables

**Table 1:** The information of test datasets.

Dataset	# car	# load of cars	Warehouse window	Time of service
RC200	30	1000	(0, 1000)	20
C300	30	800	(0, 3590)	100
R601	30	300	(0, 300)	17

## Data Availability

The data used to support the findings of this study are available from the corresponding author upon request.
